# Selective Ablation of BCL11A in Epidermal Keratinocytes Alters Skin Homeostasis and Accelerates Excisional Wound Healing In Vivo

**DOI:** 10.3390/cells11132106

**Published:** 2022-07-03

**Authors:** Nilika Bhattacharya, Arup K. Indra, Gitali Ganguli-Indra

**Affiliations:** 1 Department of Pharmaceutical Sciences, College of Pharmacy, Oregon State University, Corvallis, OR 97331, USA; bhattani@oregonstate.edu; 2 Department of Biochemistry and Biophysics, Oregon State University, Corvallis, OR 97331, USA; 3 Linus Pauling Science Center, Oregon State University, Corvallis, OR 97331, USA; 4 OHSU Knight Cancer Institute, Oregon Health & Science University (OHSU), Portland, OR 97239, USA; 5 Department of Dermatology, OHSU, Portland, OR 97239, USA

**Keywords:** epidermal permeability barrier, excisional wound healing, In Vivo, epidermal homeostasis, B-cell CLL/lymphoma 11 A (BCL11A), chicken ovalbumin upstream promoter transcription factor (COUP-TF) interacting protein 1, transcription factor, cell/non-cell autonomous, interfollicular epidermis, hair follicle, epidermis specific deletion, re-epithelialization, keratinocyte activation, differentiation

## Abstract

Transcriptional regulator BCL11A plays a crucial role in coordinating a suite of developmental processes including skin morphogenesis, barrier functions and lipid metabolism. There is little or no reports so far documenting the role of BCL11A in postnatal adult skin homeostasis and in the physiological process of tissue repair and regeneration. The current study establishes for the first time the In Vivo role of epidermal BCL11A in maintaining adult epidermal homeostasis and as a negative regulator of cutaneous wound healing. Conditional ablation of *Bcl11a* in skin epidermal keratinocytes (*Bcl11a^ep−/−^*mice) enhances the keratinocyte proliferation and differentiation program, suggesting its critical role in epidermal homeostasis of adult murine skin. Further, loss of keratinocytic BCL11A promotes rapid closure of excisional wounds both in a cell autonomous manner likely via accelerating wound re-epithelialization and in a non-cell autonomous manner by enhancing angiogenesis. The epidermis specific *Bcl11a* knockout mouse serves as a prototype to gain mechanistic understanding of various downstream pathways converging towards the manifestation of an accelerated healing phenotype upon its deletion.

## 1. Introduction

Skin, serving as the body’s first line of defense and a protective barrier, is constantly challenged with external insults that often compromises its integrity [1]. The role of skin as a “crucial stress organ” is well established. Previous studies have shown the ability of skin to produce multiple systemic factors that, when coupled with their signaling through a wide range of skin resident receptors, can serve as a cutaneous equivalent of the hypothalamus-pituitary-adrenal (HPA) axis to regulate local stress response [2]. A well-orchestrated intra- and intercellular communication triggered upon such damage to skin is the key towards effective repair and subsequent re-establishment of tissue homeostasis [3,4]. The skin is the hub of diverse cell types populating various layers, with each exhibiting specialized functionality in the physiological maintenance of skin homeostasis and trauma handling [5]. Additionally, the high accessibility and ease associated with skin sampling process make it one of the best models to study tissue repair and regeneration [6].

The skin predominantly comprises of three layers: the outermost epidermis, the lower dermis and the innermost subcutaneous tissue or the hypodermis [7,8]. The basement membrane forms the boundary between the epidermis and dermis and possesses various specialized structures such as the hair follicles (HF) and the sweat glands (SG). Reciprocal communication between the resident cell types across various layers are central towards proper skin development, homeostasis and repair [5,9]. Cutaneous wound healing is a dynamic multi-phase process involving spatial and temporal synchronization of multiple cell types, growth factors, chemokines and cytokines to facilitate repair and architecture restoration [10,11,12,13,14,15,16,17,18]. The coordinated regulation of pivotal target genes in distinct cell types by multiple transcription factors (TF) is indispensable for timely commencement, transition and smooth propagation of this multi-stage cascade to generate favorable physiological outcomes [19,20,21,22].

One such critical TF is chicken ovalbumin upstream promoter transcription factor (COUP-TF) interacting protein 1 {(CTIP1), also referred to as B-cell CLL/lymphoma 11 A (BCL11A) [23,24,25,26]. It is a C2H2 zinc finger transcriptional factor sharing high functional homology with CTIP2/BCL11B [23,24,27]. CTIP1/BCL11A interacts with COUP-TF, and Retinoid X receptor (RXR)-COUP-TF interactions have been shown to regulate retinoic acid signaling [28]. RXR mediated activation is fully repressed by COUP-TF possibly via its interactions with CTIP1/BCL11A and/or CTIP2/BCL11B. *Bcl11a* gene has five exons with exon3 and exon4 encoding up to 75% of the protein coding sequence including the DNA binding domain [26,29]. It is highly conserved, with human *Bcl11a* gene exhibiting up to 95% homology with the mouse, chicken, and Xenopus *Bcl11a* gene [27]. *Bcl11a* is a crucial developmental gene coordinating a host of developmental processes ranging from central nervous system (CNS) development to lymphopoiesis. Mice harboring germline deletion of *Bcl11a* (*Bcl11a^−/−^*) exhibit perinatal lethality, highlighting its relevance during embryogenesis [29]. A study by Li et al. in 2017 established the role of BCL11A as a critical skin morphogenesis regulator with (*Bcl11a^−/−^*) mice exhibiting Epidermal Permeability Barrier (EPB) defects, compromised skin differentiation and altered skin lipid composition [29]. In this current study, we will refer CTIP1/BCL11A as BCL11A and *Bcl11a* corresponding to the protein and gene, respectively. 

To further understand the role of BCL11A in adult skin homeostasis and in associated physiological processes, we generated a selective knockout (KO) mouse model harboring homozygous floxed *Bcl11a* allele (*Bcl11a^L2/L2^*), which in the presence of Cre-recombinase driven by K14 promoter (K14-*Cre*), undergoes conditional deletion in the epidermal keratinocytes and in the epithelium of hair follicles. We found that BCL11A has a restricted expression in adult murine skin, mostly within the HFs and along the interfollicular epidermis (IFE). Epidermis specific *Bcl11a KO mice* (*Bcl11a^ep−/−^*) exhibited altered epidermal homeostasis and enhanced expression of key epidermal proliferation and differentiation markers in normal adult skin. Further, *Bcl11a^ep−/−^ mice* displayed accelerated wound healing kinetics in an excisional wound healing model potentially via exercising its influence both in a cell and a non-cell autonomous manner. 

Taken together, this study describes a new and paradoxical role of epidermal BCL11A as a negative regulator of cutaneous excisional wound healing In Vivo, in contrast to its previously reported role as a positive regulator of skin homeostasis during development [29]. 

## 2. Materials and Methods

### 2.1. Mice

The generation of *Bcl11a* floxed mice (*Bcl11a^L2/L2^*) containing LoxP flanked exons 3 and 4 of the *Bcl11a* locus, which jointly encode ~75% of the BCL11A protein coding sequence including the DNA binding domain, was completed in an earlier study [29]. The *Bcl11a^L2/l2^* mice that was previously generated [29] were then crossed with a K14-*Cre* transgenic mice line [30] (JAX stock #004782) to generate a line having *Bcl11a* selectively ablated in the keratinocytes (epidermis specific knockout; K14*Bcl11a^ep−/−^* or *Bcl11a^ep−/−^*). *Bcl11a^ep−/−^* mice were found to exhibit no gross phenotypic changes, showed normal feeding status, survived into adulthood, and were fertile. Mice were sheltered in our approved university animal facility with 12 h light cycles and supplied with food and water ad libitum. A combination of both male and female mice was used for all the studies.

### 2.2. Tail DNA Isolation and Polymerase Chain Reaction (PCR) Genotyping

The tail tipping or biopsy procedure was performed as described in the investigator’s approved IACUC protocol. Tail biopsies (1–2 mm) were collected from 10–17-day-old mice. Each harvested mouse tail was digested for 6 h at 55 °C and 650 revolutions per minute (RPM) in 500 µL of PK buffer {200 mM NaCl, 1% SDS, 50 mM Tris-HCl (pH 7.5), 5 mM EDTA (pH 8.0)} plus 0.2 mg/mL proteinase K in a thermomixer (Eppendorf 5350). After digestion, tail DNA extraction was performed employing chloroform and ethanol precipitation method followed by PCR genotyping using specific primers (listed in Table S1) for the simultaneous detection of *Cre* and *Bcl11a* excision allele (*L-*) [29].

### 2.3. Murine Tail Skin Epidermis-Dermis Separation

P39/40 adult mice were euthanized by CO_2_ inhalation as per the IACUC guidelines. Following the removal of the tail bone, the whole tail skin was washed in sterile 1X Phosphate Buffered Saline (PBS) to get rid of the blood stains. It was then suspended in 20 mM Na_2_EDTA and 15 mM sodium phosphate buffer solution (pH 7.2) in a 15 mL conical tube. The tubes were subsequently mounted on a tube rotator for 4 h at 37 °C. At the end of 4 h, each of the tail samples were washed in sterile 1X PBS (3 times for 5 min each) on a rocker followed by their storage in 200 µL of 20 mM Hepes, 250 mM NaCl, 2 mM EDTA, 10% glycerol, 1 M NaF, 2 mM Hemin Chloride, 400 mM NEM, 100 mM PMSF, 100× PIC and 10% SDS lysis buffer (TD250+/SDS) at −80 °C.

### 2.4. Wound Healing Assay

*Bcl11a^L2/L2^* and *Bcl11a^ep−/−^* mice were used for the study. Two 5 mm full thickness wounds were generated on the dorsal skin of 11–19-week-old adult mice (sex combined) using a 5 mm punch biopsy (Integra Miltex). The wounds were imaged digitally on Day 1 and Day 8 with the wound closure rate being analyzed by measuring the diameter of each wound using VWR Traceable^R^ Carbon Fiber Calipers 6in (36934154) on alternate days till Day 10 depending on the healing rate. Two independent experiments were preformed comprising a total of 8 subjects per group (4 females and 4 males) to study gender differences and considering “sex” as a crucial biological variable. The Day 8 and Day 10 final samples collection involved the excision of the complete wound tissue with 2 mm margin on either side, which was then bisected into two halves. One half of it was immediately snap-frozen in liquid nitrogen for future immunoblotting, while the other half was harvested for histology and immunohistochemical analysis. 

### 2.5. Immunoblotting

The harvested tissue samples were initially shredded into small chunks that were then subjected to lysis by sonicating them in a denaturing TD250+/SDS buffer for 6–8 cycles (depending on the sample type), each lasting for 10 s at power level 4 at room temperature (RT) in a sonicator {Microson^TM^ Ultrasonic Cell Disruptor (Model No: XL2000)} [31]. The lysates were then centrifuged at 13,000 RPM at RT to get rid of the DNA and the cell debris. Protein concentrations of the saved supernatants were then determined by BCA protein assay (Thermo-Fisher Scientific Pierce^TM^ BCA Protein Assay Kit, Rockford, IL, USA). Equal amounts of whole cell lysates (20–25 µg) were loaded and run on an SDS-polyacrylamide gels followed by their transfer to nitrocellulose membranes. The blots were then blocked for an hour with 5% nonfat dry milk or Bovine Serum Albumin (BSA) followed by an overnight incubation with specific primary antibodies (listed in Table S2). Horseradish peroxidase conjugated secondary antibodies were used and blots were developed using Amersham^TM^ ECL Prime Western Blot Detection Reagent. Immunoblot quantification was performed using free Image J software available from National Institute of Health (Bethesda, MD, USA) website. 

### 2.6. Histology

The dorsal skin biopsies harvested from various regions at respective time-points from the *Bcl11a^L2/L2^* v/s *Bcl11a^ep−/−^* mice were initially fixed overnight at 4 °C in 4% paraformaldehyde, followed by subsequent treatment with a graded series of alcohol and xylene before final embedding in paraffin [32]. 5 µm thick paraffin sections were cut onto VWR Superfrost Plus Slides using D554X microtome blades (C.L. Sturkey, Lebanon, PA, USA) on RM2255 microtome (Leica Microsystems, Wetzlar, Germany). Hematoxylin and Eosin (H and E) staining were performed following deparaffinization of 5 µm thick sections through a graded series of xylene and ethanol as described for histological analysis [32].

### 2.7. Immunohistochemistry (IHC)

Slides containing 5 µm thick paraffin skin sections were rehydrated using graded series of xylene, ethanol and water followed by microwave mediated antigen retrieval in citrate buffer (pH 6.0) at power level 8 for 5 × 5′. The slides were then cooled for 20 min followed by washing with 0.05% tween supplemented PBS (PBST) to permeabilize the tissue sections. Blocking was performed for 30 min at RT in 10% normal goat serum (NGS) in PBST followed with incubation with respective primary antibodies at the appropriate dilutions (listed in Table S3) overnight at 4 °C. The subsequent day, following a series of washes with PBST, slides were incubated with secondary antibodies conjugated with CY3 or CY2 fluorophores (listed in Table S3) at RT for 2 h. All slides were counterstained with 0.2 ng/mL 4′,6-diamidino-2-phenylindole (DAPI) in PBST at RT for 10 min. Slides were finally dehydrated and mounted with dibutyl phthalate in xylene (VWR).

### 2.8. Imaging

H and E images were captured at 2× and 10× magnification using Keyence BZ-X700 fluorescence microscope. Fluorescence imaging was performed at either 10× or 20× (objective magnification) depending upon a specific molecular marker, using Zeiss AXIO Imager.Z1 with a digital AxioCam HRm, which were then processed using AxioVision 4.8 (Carl Zeiss, Oberkochen, Germany) and Adobe Photoshop 2020 (Adobe, San Jose, CA, USA). 

### 2.9. IHC Analysis and Quantification

All analysis and quantifications were performed using ImageJ software freely available from the NIH, USA. Depending on the nature of the marker (nuclear or cytoplasmic), the quantification method was optimized to capture the true expression of the various markers across the specific regions or the compartments of interest. Multiple fields of view (or individual images) from each biological replicate were analyzed by two independent investigators to ensure accurate representation of the real In Vivo situation.

To quantify the expression of cytoplasmic markers {Keratin 6 (K6), Keratin 14 (K14) and Keratin 10 (K10)} at baseline level and across various stages of wounding, we used ImageJ. All the raw images were initially adjusted for background and intensity using Adobe Photoshop 2020. Following that, each individual images were converted to RGB stack using ImageJ to split different channels into separate windows (such as all DAPI stained nucleated cells in one and cytoplasmic marker-stained cells in the other). 

For quantification of marker with cytoplasmic staining, regions of interest (ROI) were manually drawn around a particular skin compartment (epidermis in this case) to understand the localized compartment specific expression pattern of the marker of interest. Identical parameters such as lower and upper threshold level were set across all the individual images to correctly detect the difference between the sample groups, not incorporating errors resulting from under or overexposure of the individual images. Ultimately employing the ROIs, area fraction positive for various markers was computed for each individual image, essentially depicting what fraction of the total epidermis is expressing the marker in each field of view (FOV).

For quantification of active proliferation and nuclear marker {Proliferating Cell Nuclear Antigen (PCNA)}, using the ImageJ cell counter tool, the total number of nucleated cells (DAPI labelled cells) and number of PCNA positive cells (co-labelled with DAPI and PCNA) were respectively counted. The number of PCNA positive cells were then expressed as a percentage of the total number of the nucleated cells (restricted to the epidermal compartment) to determine epidermis specific alteration in the expression of PCNA across the various stages.

ImageJ was also used for computing the length of individually distinguishable myofibroblasts from α-SMA staining. The scale was first set employing a blank 20× image overlayed with the 50 µm scale bar. The straight-line icon was then selected and stretched over the 50 µm scale bar to encompass the entire length. The scale was finally set using the “Set Scale” tool under the “Analyze” tab with 50 µm corresponding to 624.1154 pixels. The “global” option was enabled in the “Set Scale” window to make sure all the images open with the same settings. Following that, the image of interest was opened in the appropriate filter channel corresponding to the stain (in this case CY3) and the straight-line icon was extended across various individual myofibroblasts dispersed across the FOV to measure their corresponding length in µm.

### 2.10. Immunoblot Analysis and Quantification

Imaging of immunoblots was performed using MY ECL IMAGER (Catalog No: 62236X) at multiple exposures. The density of the bands from the respective immunoblots were quantified using freely downloadable ImageJ software (NIH, Bethesda, MD, USA) and normalized with respect to β-actin.

### 2.11. Statistical Analysis

Statistical significance was calculated using unpaired *t*-test employing GraphPad Prism5 software.

## 3. Results

### 3.1. Confirmation of Epidermis Specific Bcl11a Excision in Bcl11a^ep−/−^ Mice

**Figure 1:**
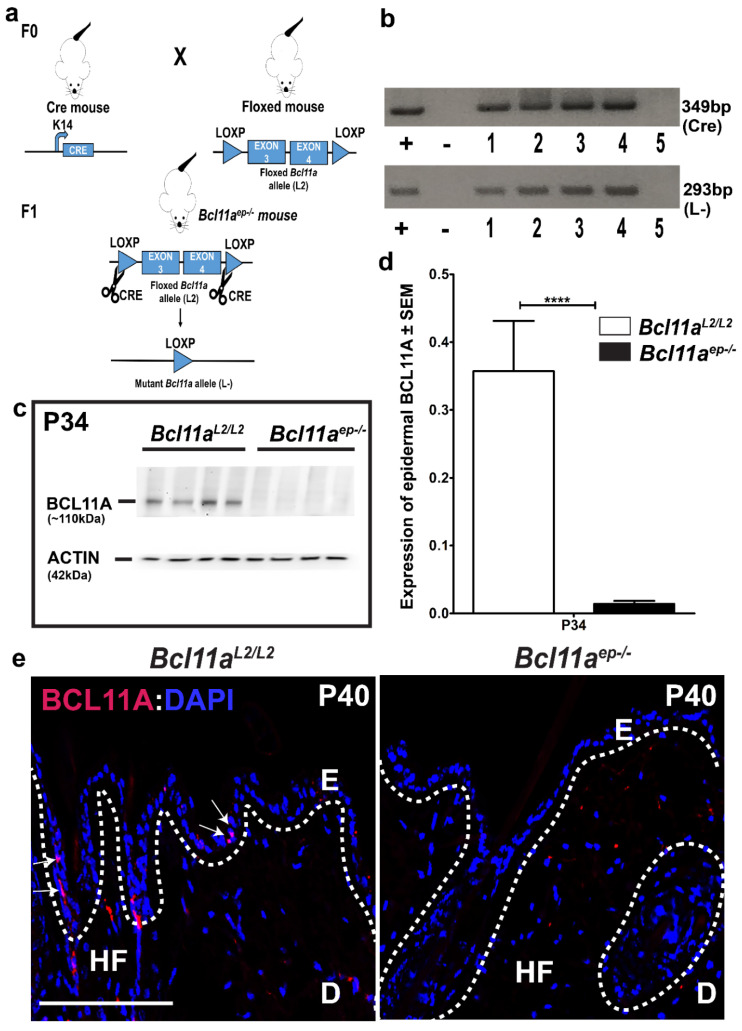
Confirmation of epidermal specific *Bcl11a* excision in *Bcl11a^ep−/−^* mice. (**a**) Schematic representation of Cre-LoxP mediated generation of *Bcl11a^ep−/−^* mice. (**b**) PCR amplification of Cre and L- (excision) alleles on tail DNA extracted from *Bcl11a^L2/L2^* and *Bcl11a^ep−/−^* mice. “+” and “−” indicates positive and negative control with PCR being performed on isolated DNA from pre-confirmed *Bcl11a^ep−/−^* mice and Milli-Q water, respectively. 1,2,3,4,5 represent mouse ID numbers (either *Bcl11a^L2/L2^* or *Bcl11a^ep−/−^*) from which the tail tip was harvested for subsequent DNA extraction and PCR. (**c**) Immunoblot analysis of epidermal protein extracts from postnatal day 34 (P34) tail skin samples using anti-BCL11A monoclonal antibody. β-actin was used as the internal control. (**d**) Bar graph quantification of P34 epidermal BCL11A expression in wildtype (*Bcl11a^L2/L2^*) versus mutant (*Bcl11a^ep−/−^*) mice. (**e**) IHC of 5-μm thick dorsal skin sections with anti-BCL11A antibody (red) in *Bcl11a^L2/L2^* versus *Bcl11a^ep−/−^* mice at P40. All sections were counterstained with DAPI (blue). White dotted lines separate E from D and outline the HFs. White arrowheads indicate nuclear localization of BCL11A in the IFE and HF. D—Dermis; E—Epidermis; IFE—Interfollicular epidermis; HF—Hair follicle. Scale bar = 50 µM. N = 4 (for (**c**)) and N = 2 (for (**e**)), where N represents the number of animals of each genotype (*Bcl11a^L2/L2^* or *Bcl11a^ep−/−^*) being included in the study. 10–12 field of views (FOVs) were captured per sample per genotype for calculating the significance. **** *p* < 0.001. * denotes the degree of statistical significance.

*Bcl11a^L2/L2^* mice were crossed with a K14-Cre transgenic mice to generate a new line in which *Bcl11a* was selectively ablated in the epidermal keratinocytes (Figure 1a) [29,30]. Conditional deletion of *Bcl11a* at the gene level was initially confirmed through PCR genotyping. Two specific set of primers were used to detect the concurrent presence of *Cre* and excised *Bcl11a* allele (*L-*) (generated upon Cre mediated recombination) in *Bcl11a^ep−/−^* mice (Figure 1b) [29]. To further confirm epidermis specific BCL11A excision at the protein level, immunoblotting was performed on tail epidermal extracts harvested from postnatal day 34 (P34) *Bcl11a^L2lL2^* vs *Bcl11a^ep−/−^* mice. A specific band corresponding to BCL11A with a molecular weight of approximately 110kDa was detected in the *Bcl11a^L2/L2^* mice in contrast to no detectable expression in the *Bcl11a^ep−/−^* mice (Figure 1c,d). Additionally, to validate BCL11A excision across all epidermal compartments (HF and IFE), immunohistochemistry was performed on P40 dorsal skin samples to initially characterize the expression pattern of BCL11A in wildtype adult skin and thereby confirm its targeted deletion across epidermis in *Bcl11a^ep−/−^* mice. A restricted nuclear BCL11A expression was detected in the IFE and HFs of adult wildtype (*Bcl11a^L2/L2^*) skin, which was completely absent in the epidermis and HFs of *Bcl11a^ep−/−^* mice (Figure 1e). The above results confirmed an efficient K14 promoter driven *Cre* mediated epidermal deletion of *Bcl11a.*

### 3.2. Altered Epidermal Homeostasis in Bcl11a^ep−/−^ Adult Skin

**Figure 2:**
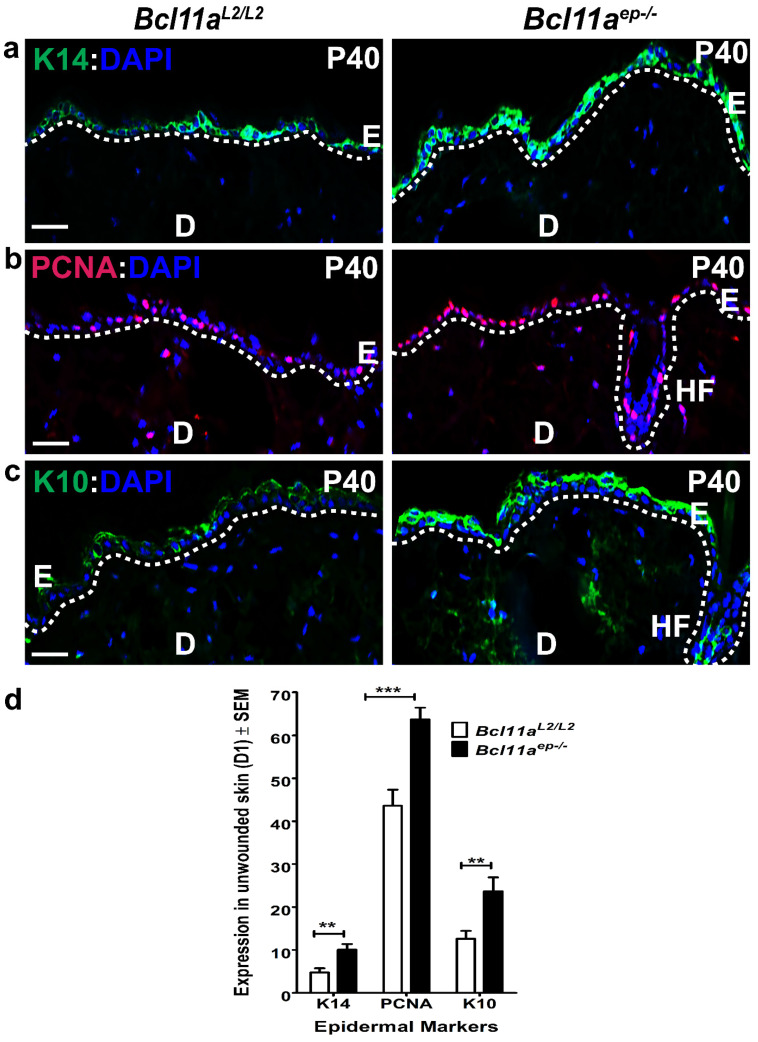
Conditional ablation of *Bcl11a* in adult epidermis leads to enhanced expression of key epidermal proliferation and differentiation markers in normal skin. (**a**–**c**) IHC of P40 dorsal skin sections with basal keratinocyte marker K14 (green), active proliferation marker PCNA (red) and early differentiation marker K10 (green) in *Bcl11a^L2/L2^* versus *Bcl11a^ep−/−^* mice. All 5 μm-thick paraffin sections were counterstained with DAPI (blue). White dotted lines separate E from D and outline the HFs. D, dermis; E, epidermis; HF, Hair follicle. Scale bar = 50 μm. (**d**) Bar graph quantification of baseline expression of epidermal markers (K14, PCNA, K10) in P40 *Bcl11a^L2/L2^* versus *Bcl11a^ep−/−^* skin. N = 2, where N represents the number of animals of each genotype (*Bcl11a^L2/L2^* or *Bcl11a^ep−/−^*) being involved in the study. 10–12 field of views (FOVs) captured per sample per genotype to determine the statistical significance. ** *p* < 0.01, *** *p* < 0.001.

We next examined the effects of conditional *Bcl11a* deletion on proliferation and differentiation and determined its involvement in maintaining the homeostasis of murine epidermis [33]. To that end, we looked at the expression status of K14 (basal proliferative keratinocyte marker), PCNA (active proliferation marker) and K10 (early differentiation marker) in P40 *Bcl11a^L2/L2^* v/s *Bcl11a^ep−/−^* mice dorsal skin samples by IHC [34]. Significant upregulation in epidermal K14, PCNA and K10 expression was observed in *Bcl11a^ep−/−^* mice as compared to the control *Bcl11a^L2/L2^* mice with the functional protein (Figure 2a,c), which was further confirmed by quantification (Figure 2d). Additional immunoblotting followed by quantification for K14, PCNA, and K10 on P39 tail epidermal extracts revealed their enhanced expression in *Bcl11a^ep−/−^* mice, thereby corroborating our IHC observations made on dorsal skin samples (Supplementary Figure S1a,b). Collectively, these results suggest that BCL11A in epidermis is critical for regulating physiological dynamics between proliferation and differentiation during skin homeostasis.

### 3.3. Accelerated Cutaneous Wound Healing in Bcl11a^ep−/−^ Mice

**Figure 3:**
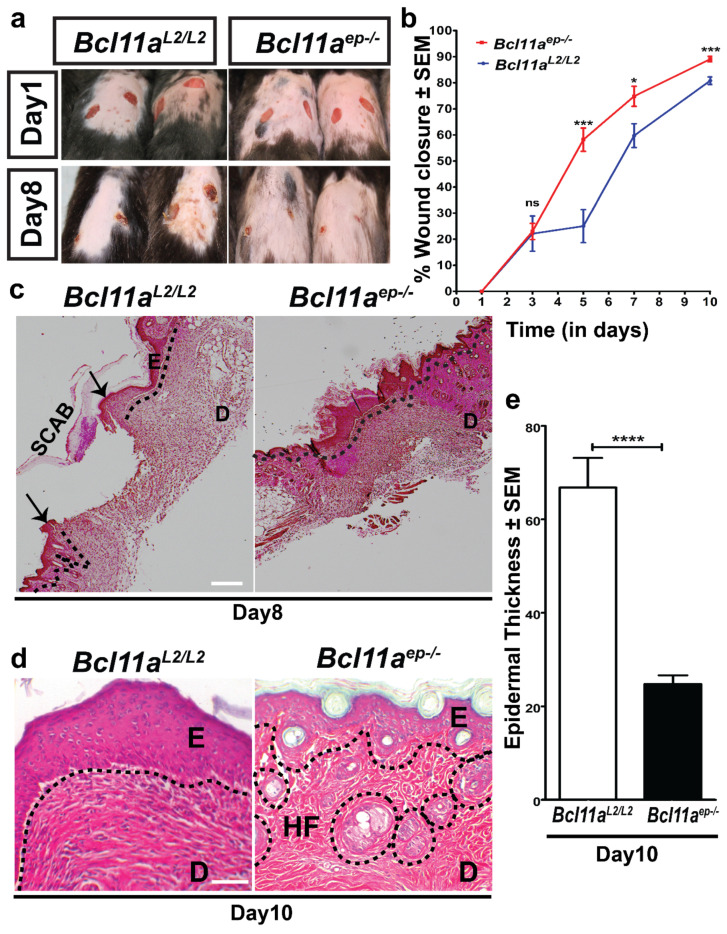
Accelerated wound healing in *Bcl11a^ep−/−^* mice. (**a**) Macroscopic images of the comparative healing dynamics of 5 mm full thickness excisional wounds in *Bcl11a^L2/L2^* versus *Bcl11a^ep−/−^* mice on Day 1 and Day 8 post wounding. (**b**) Graphical representation of the rate of wound closure at various timepoints post wounding in *Bcl11a^L2/L2^* and *Bcl11a^ep−/−^* mice. Statistical significance between the two groups at each interval were computed employing Student’s unpaired *t*-test (* *p* < 0.1, *** *p* < 0.001, ^ns^ < non-significant). (**c**) Hematoxylin and Eosin (H and E) stained images of the Day 8 post wounding samples. The black arrows indicate the two migratory epithelial tongues progressively moving towards each other to regenerate the denuded epidermis. (**d**) H and E-stained images of the Day 10 post wounding samples. The black dotted lines separate E from D and outline the HFs. Hyperproliferative epithelium; E—Epidermis; D—Dermis; HF—Hair follicle. Scale bar = 100 μm. (**e**) Bar graph quantification of the relative thickness of newly formed epidermis in *Bcl11a^L2/L2^* versus *Bcl11a^ep−/−^* mice. N = 8, where N represents the number of animals of each genotype (*Bcl11a^L2/L2^* or *Bcl11a^ep−/−^*) involved in the study. Wherever applicable, multiple fields of views (FOVs) captured per sample per genotype at each timepoint to determine the statistical significance. **** *p* < 0.0001.

Based on the prior knowledge of BCL11A as a crucial skin morphogenesis regulator [29], we hypothesized that epidermal BCL11A could be a potential regulator of cutaneous wound healing. To test that, we performed In Vivo cutaneous wound healing assay in *Bcl11a^L2/L2^* vs *Bcl11a^ep−/−^* mice as described in the material and methods section [21]. Digital and graphical analysis of the wound diameter on alternate days post wounding showed significantly higher percentage of wound closure at different days post wounding (Days 5, 7 and 10) and enhanced healing in the *Bcl11a^ep−/−^* mice in an un-stented wound healing model (Figure 3a,b). Additional histological analysis made on H and E-stained skin wound samples harvested on Days 8 and 10 post wounding revealed a clearly visible distinct boundary separating the newly formed hyperproliferative epidermis from the dermis (highlighted by a single black dotted line) in the Day 8 *Bcl11a^ep−/−^* wound bed, which signifies early re-epithelialization ((Figure 3c, right panel) [21,35]. In contrast, re-epithelialization was yet to be completed in wild type *Bcl11a^L2lL2^* wound bed (as indicated by the two black arrows pointing towards the two epithelial migratory tongues on either side of the wound bed) (Figure 3c, left panel). Histological analysis and quantification of Day 10 wound epidermal thickness revealed the existence of a persistent hyperproliferative epithelium in the *Bcl11a^L2/L2^* mice, which was significantly reduced in the *Bcl11a^ep−/−^* mice, suggesting early restoration of homeostasis (Figure 3d,e) [35,36]. Taken together, results suggest that the loss of BCL11A in the epidermis facilitates accelerated healing possibly through rapid re-epithelialization followed by quicker restoration of homeostasis.

### 3.4. Bcl11a in a Cell Autonomous Manner Promotes Accelerated Healing via Early Keratinocyte Activation, Rapid Re-Epithelialization and Onset of Differentiation

We hypothesized that epidermal *Bcl11a* deletion modulates cutaneous wound healing in a cell autonomous manner. To validate that, we performed IHC on the Day 8 wound bed samples of *Bcl11a^L2lL2^* v/s *Bcl11a^ep−/−^* mice for various epidermal markers known to be functional during different phases of healing to understand their dynamics in absence of epidermal *Bcl11a*. 

**Figure 4:**
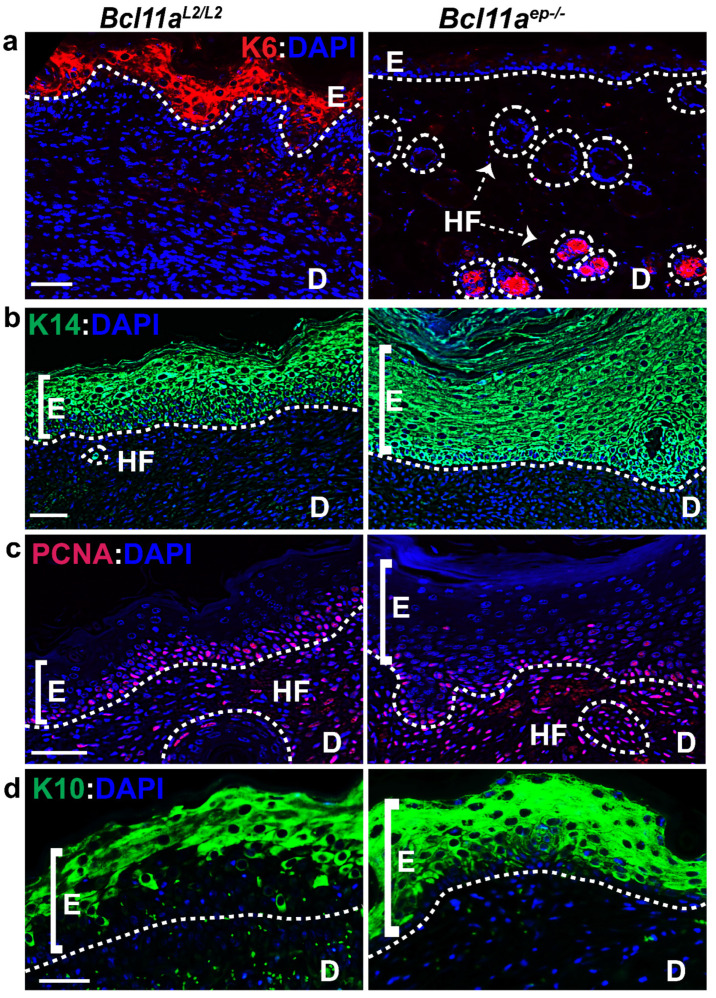
Cell autonomous role of epidermal specific *Bcl11a* ablation in promoting accelerated healing by early keratinocyte activation, re-epithelialization, and rapid onset of differentiation (Day 8). (**a**–**d**) IHC analysis of activated keratinocyte marker K6 (red), epidermal proliferative basal keratinocyte marker K14 (green), active proliferation marker PCNA (red), and early differentiation marker K10 (green) expression in WBAE on Day 8 post wounding samples from *Bcl11a^L2/L2^* versus *Bcl11a^ep−/−^* mice. All sections were counterstained with DAPI (blue). White dotted lines separate E from D and outline the HFs. D—Dermis; E—Epidermis; HF—Hair follicle; WBAE—Wound Bed Adjacent Epidermis. Scale bar = 50 μm. N = 4, where N represents the number of animals of each genotype (*Bcl11a^L2/L2^* or *Bcl11a^ep−/−^*) involved in the study. Multiple fields of views (FOVs) captured per sample per genotype for statistical analysis.

**Figure 5:**
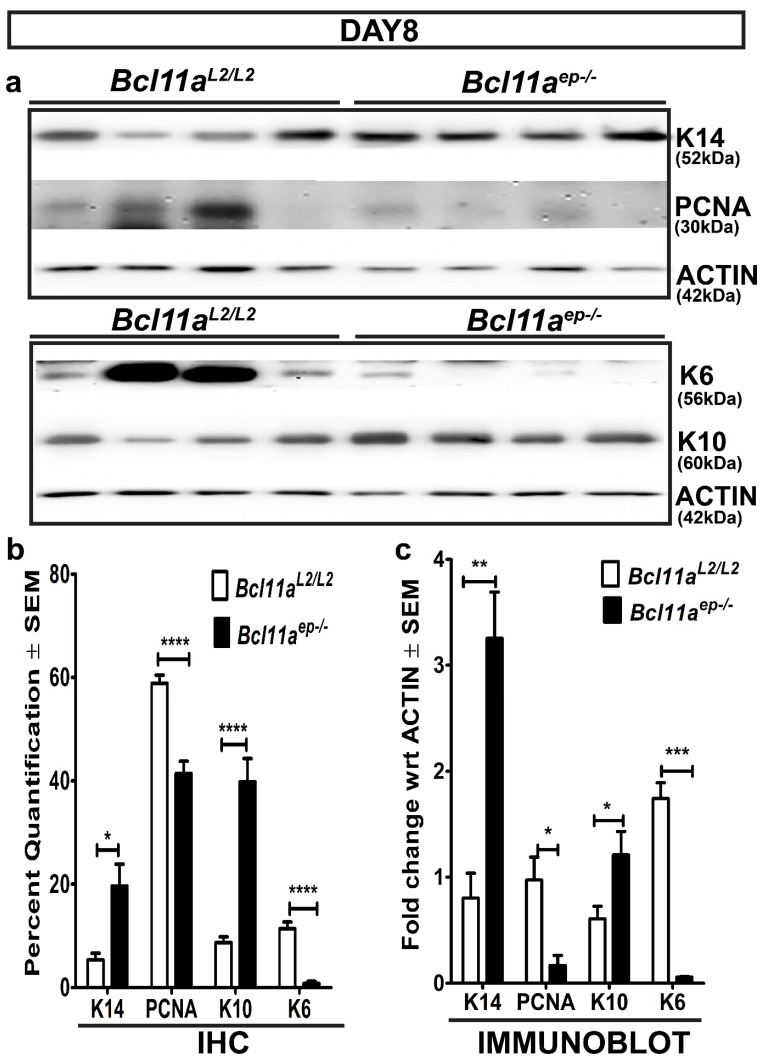
Epidermis specific *Bcl11a* deletion alters expression profile of key markers of epidermal homeostasis on Day 8 post wounding. (**a**) Immunoblot analyses of basal keratinocyte marker (K14), active proliferation marker (PCNA), activated keratinocyte marker (K6), and early differentiation marker (K10) expression in the dorsal skin of *Bcl11a^L2/L2^* versus *Bcl11a^ep−/−^* adult mice on Day 8 post wounding. β-actin was used as the internal control. (**b**) IHC quantification displaying the Day 8 post wounding expression status of epidermal markers (K14, PCNA, K10 and K6) in *Bcl11a^L2/L2^* versus *Bcl11a^ep−/−^* mice. (**c**) Immunoblot quantification showing the relative fold change in the expression status of epidermal markers (K14, PCNA, K10 and K6) in *Bcl11a^L2/L2^* versus *Bcl11a^ep−/−^* Day 8 wounds. N = 4, where N represents the number of animals of each genotype (*Bcl11a^L2/L2^* or *Bcl11a^ep−/−^*) being involved in the study. All western blots were repeated 2–3 times. * *p* < 0.1, ** *p* < 0.01, *** *p* < 0.001, **** *p* < 0.0001.

Physiological expression of the activated keratinocyte marker (K6) is primarily localized along the length of the HF with some expression across the supra-basal layer of the eccrine sweat duct [37]. However, injury stimulates K6 expression in the supra-basal keratinocytes adjacent to the wound edges ahead of their migration to the wound site and persistently throughout re-epithelialization [21,37,38,39,40,41]. Immunohistochemical characterization of K6 on the Day 8 samples showed intense expression across wound bed adjacent epidermis (WBAE) in *Bcl11a^L2/L2^* control mice, but more restricted expression within the HFs in *Bcl11a^ep−/−^* mice (Figure 4a and Figure 5b). It was further authenticated by immunoblot analysis of the Day 8 wounds, which showed a significant downregulation of K6 expression in *Bcl11a^ep−/−^* mice, suggesting prompt resolution of the initial activation phase of healing (Figure 5a,c).

Healing involves phases of rapid proliferation during re-epithelialization ensued upon by active differentiation for restoration of homeostasis. We therefore looked at the expression of proliferation marker PCNA and keratinocyte basal cell marker K14 to gain insight into the re-epithelialization process (Figure 4b,c and Figure 5b) [21,34,35,37,42,43,44]. Immunohistochemical characterization of PCNA across WBAE on the Day 8 samples revealed significant downregulation of PCNA expression in *Bcl11a^ep−/−^* mice relative to the *Bcl11a^L2lL2^* control mice (Figure 4c and Figure 5b). However, we observed an increase in K14 expression across WBAE in *Bcl11a^ep−/−^* mice compared to the control mice at Day 8, signifying an advanced stage of re-epithelization, potentially including all epithelial cells contributing to the newly regenerated epidermis (Figure 4c and Figure 5b). Further validation by immunoblot analysis showed similar downregulation and upregulation of active proliferation marker PCNA and basal proliferative keratinocyte marker K14 expression, respectively, in the *Bcl11a^ep−/−^* wounds in comparison to the control wounds (Figure 5a,c). 

Keratin 10 (K10) is an early marker of differentiation, and its expression is restricted to the supra-basal layer of the epidermis [45]. During the later stages of re-epithelialization, the migrating epidermal keratinocytes gradually start expressing K10, indicating their gradual commitment to differentiation which ultimately leads to homeostasis [15,21,46]. Immunohistochemical characterization of the Day 8 samples revealed the sparse K10 expression in *Bcl11a^L2/L2^* control mice justified its normal commitment to differentiation during re-epithelialization (Figure 4d and Figure 5b) [29,47]. On the other hand, we noted elevated expression of K10 along the supra-basal layer of the WBAE in *Bcl11a^ep−/−^* mice, thereby indicating early commitment to differentiation and rapid re-establishment of the normal differentiation program (Figure 4d and Figure 5b). Further corroboration came from the Day 8 wound immunoblot analysis showing a significant upregulation of K10 expression in *Bcl11a^ep−/−^* mice relative to the control wounds, indicating rapid re-epithelialization and onset of differentiation (Figure 5a,c). Overall, the above results depicted the impact of epidermis specific *Bcl11a* ablation in accelerating the epidermal proliferation and differentiation kinetics in the wound bed and adjacent regions during healing to achieve accelerated wound closure.

### 3.5. Epidermal Bcl11a Deletion Promotes Advanced Angiogenesis and Myofibroblast Mediated Wound Contraction in a Non-Cell Autonomous Manner

We hypothesized that epidermal *Bcl11a* deletion might have the potential to modulate cutaneous wound healing in a non-cell autonomous manner. Two key processes central to efficient wound closure are angiogenesis and fibroplasia, neither of which involves epidermal keratinocytes [15]. Hence deciphering the effects of epidermal *Bcl11a* ablation on these events would enable us to determine its non-cell autonomous role in facilitating healing. 

**Figure 6:**
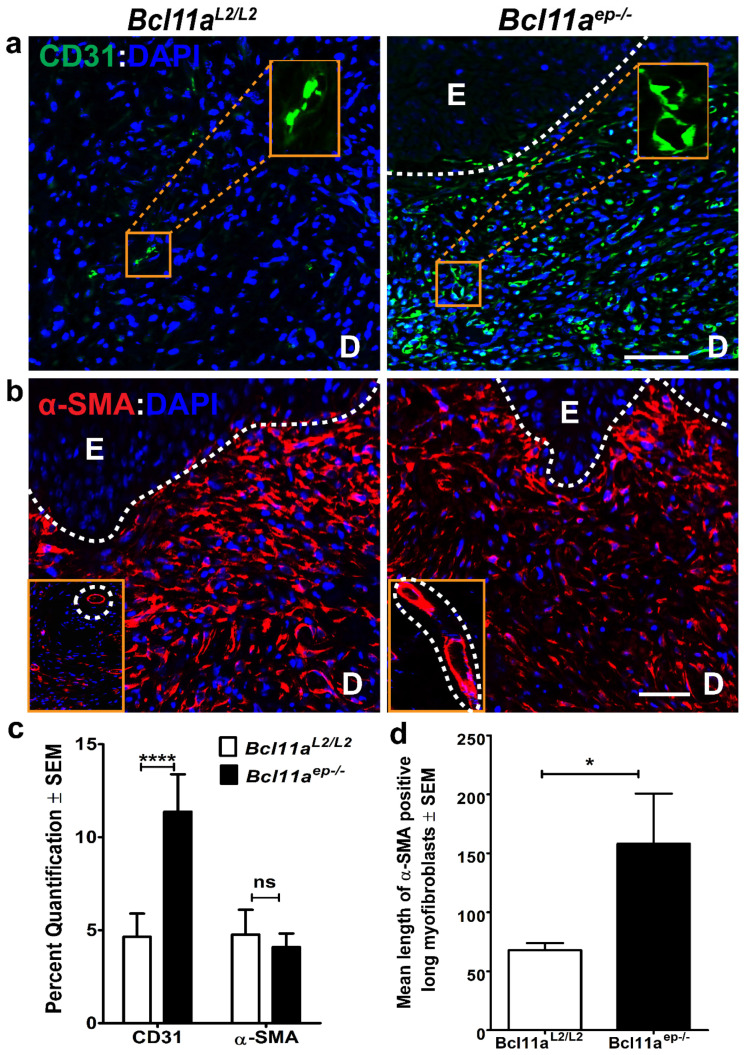
Non-cell autonomous role of epidermal *Bcl11a* deletion in facilitating healing through advanced angiogenesis and myofibroblast maturation (Day 8). (**a**,**b**) IHC analysis of endothelial cell marker CD31 (green) and myofibroblast marker α-SMA (red) expression in WBD on Day 8 post wounding samples from *Bcl11a^L2/L2^* versus *Bcl11a^ep−/−^* mice. All sections were counterstained with DAPI (blue). White dotted lines separate E from D and outline the HFs. The insets respectively show magnified version of blood vessels formed by endothelial cells (**a**) and mature myofibroblasts (**b**). D—Dermis; E—Epidermis; HF—Hair follicle; WBD-Wound Bed Dermis. Scale bar = 100 μm. (**c**) Bar graph quantification displaying the Day 8 post wounding expression status of dermal markers (CD31 and α-SMA) in *Bcl11a^L2/L2^* versus *Bcl11a^ep−/−^* mice. (**d**) Bar graph quantification highlighting the difference in the mean length (in μm) of mature myofibroblasts in the Day 8 wound bed of *Bcl11a^L2/L2^* versus *Bcl11a^ep−/−^* mice. N = 4, where N represents the number of animals of each genotype (*Bcl11a^L2/L2^* or *Bcl11a^ep−/−^*) being involved in the study. Multiple fields of views (FOVs) captured per sample per genotype to determine the statistical significance. **** *p* < 0.0001, * *p* < 0.1, ^ns^ < non-significant.

Neovascularization or angiogenesis is critical for wound healing to restore the vascular perfusion to the denuded area and thereby enable formation of the granulation tissue [15]. Immunohistochemical characterization of the expression of CD31 [31], an endothelial cell marker in *Bcl11a^L2/L2^* mice, showed dispersed presence of individual endothelial cells in the Day 8 wound bed, indicating their initial recruitment. In contrast, the abundance and size of blood vessels was significantly increased in the *Bcl11a^ep−/−^* mice (Figure 6a,c), which signified advanced angiogenesis.

With the emergence of the new blood vessels, resident fibroblasts proliferate and invade the clot to form contractile granulation tissue [15]. Further maturation of these fibroblasts to myofibroblasts ultimately mediate wound contraction by drawing the wound margins together. Alpha smooth muscle actin (α-SMA) is a universal marker of myofibroblasts [48]. Immunohistochemical analysis of α-SMA positive myofibroblast abundance in the Day 8 wound bed samples revealed a trend of downregulation in the *Bcl11a^ep−/−^* mice (Figure 6b,c). However, this difference wasn’t significant. Further, activated myofibroblasts showed an elongated and stellate morphology in the mutant group in contrast to the circular morphology exhibited by the control (shown in insets). Additional quantification computing the mean length of individual mature myofibroblasts showed significant increase in absence of epidermal *Bcl11a* (Figure 6d), indicating rapid maturation and subsequent contraction to facilitate healing. Taken together, these results indicate that epidermal *Bcl11a* in a non-cell autonomous manner promotes healing through advanced angiogenesis and rapid myofibroblast mediated wound contraction and remodeling [49].

### 3.6. Rapid Re-Establishment of Epidermal Homeostasis in the Later Stages of Healing in Bcl11a^ep−/−^ Mice

We further postulated that epidermal *Bcl11a* deletion might facilitate early restoration of skin homeostasis during later stages of healing by restoring the expression of the key epidermal markers to normal physiological levels [33]. To that end, we examined the expression of keratinocyte activation and epidermal proliferation and differentiation markers, including K6, K14, PCNA and K10 in the Day 10 *Bcl11a^L2/L2^* v/s *Bcl11a^ep−/−^* wound bed samples by IHC and immunoblot analysis. 

**Figure 7:**
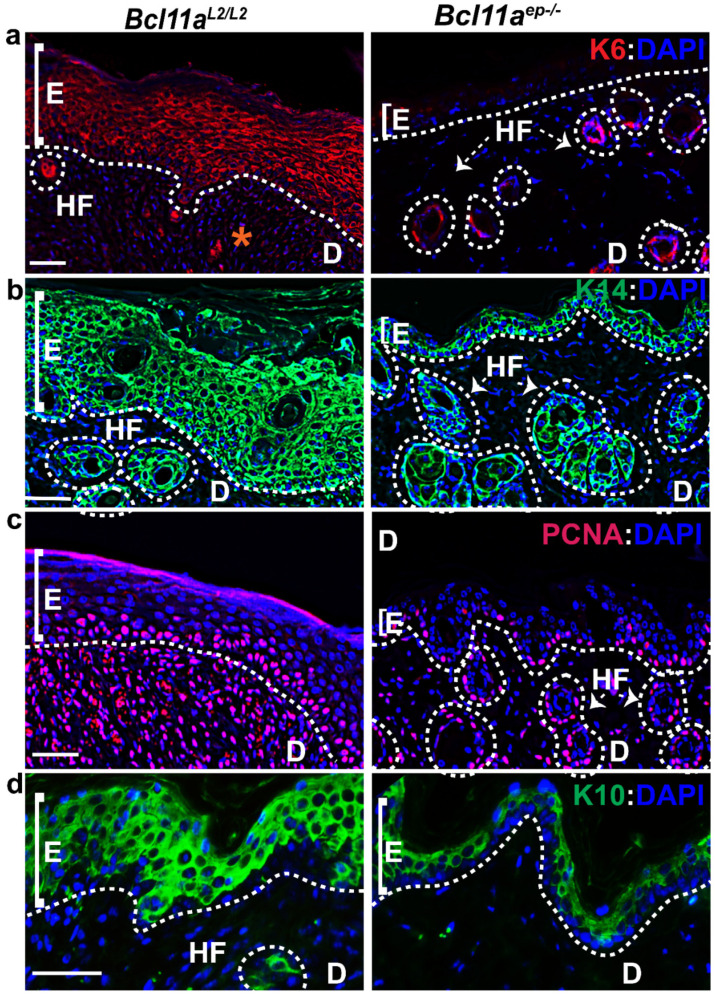
*Bcl11a^ep−/−^* mice exhibits rapid re-establishment of epidermal homeostasis through restored expression of epidermal markers during late stages of wound healing (Day 10). (**a**–**d**) IHC analysis of activated keratinocyte marker K6 (red), re-epithelialization marker K14 (green), active proliferation marker PCNA (red), and early differentiation marker K10 (green) expression in WBAE on Day 10 post wounding samples from *Bcl11a^L2/L2^* versus *Bcl11a^ep−/−^* mice. All sections were counterstained with DAPI (blue). White dotted lines separate E from D and outline the HFs. D—Dermis; E—Epidermis; HF—Hair follicle; WBAE—Wound Bed Adjacent Epidermis. Scale bar = 50 μm. N = 4, where N represents the number of animals of each genotype (*Bcl11a^L2/L2^* or *Bcl11a^ep−/−^*) involved in the study. Multiple Field of views (FOVs) captured per sample per genotype to determine the statistical significance. “*” in the Figure 7a (left panel) indicates non-specific staining.

**Figure 8:**
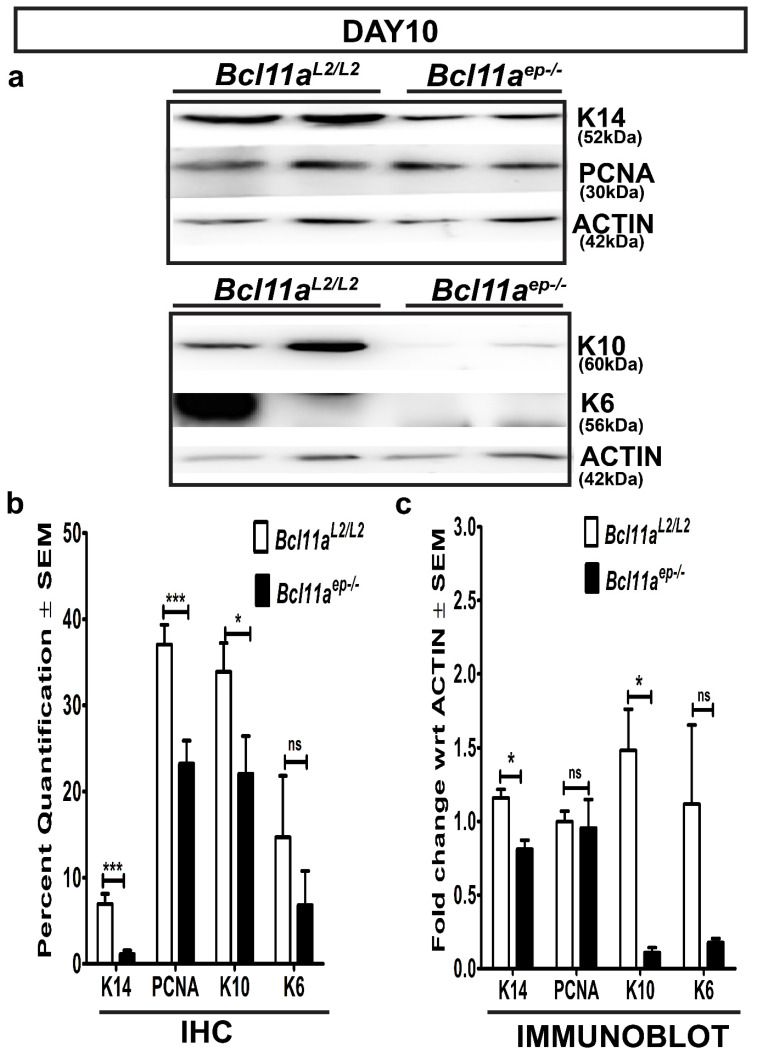
Epidermis specific *Bcl11a* deletion restores expression profile of epidermal markers to physiological levels on Day 10 post wounding. (**a**) Immunoblot analyses of basal keratinocyte marker (K14), active proliferation marker (PCNA), activated keratinocyte marker (K6), and early differentiation marker (K10) expression in the dorsal skin of *Bcl11a^L2/L2^* versus *Bcl11a^ep−/−^* adult mice on Day 10 post wounding. β-actin was used as the internal control. (**b**) IHC quantification displaying the Day 10 post wounding expression status of epidermal markers (K14, PCNA, K10 and K6) in *Bcl11a^L2/L2^* versus *Bcl11a^ep−/−^* mice. (**c**) Immunoblot quantification showing the relative fold change in the expression status of epidermal markers (K14, K10 and K6) in *Bcl11a^L2/L2^* versus *Bcl11a^ep−/−^* Day 10 wounds. N = 4, where N represents the number of animals of each genotype (*Bcl11a^L2/L2^* or *Bcl11a^ep−/−^*) being involved in the study. Western blots were repeated 2–3 times. * *p* < 0.1, *** *p* < 0.001, ^ns^ < non-significant.

Immunohistochemical characterization of the Day 10 wound bed samples revealed elevated expression of activated keratinocyte marker (K6), proliferation marker PCNA, proliferating keratinocyte basal cell marker K14 and early differentiation marker K10 in the epidermis of the *Bcl11a^L2/L2^* control skin (Figure 7 and Figure 8b). In contrast, a significant downregulation of expression of K6, K14, PCNA) and K10 was noted in the D10 skin wound biopsies from *Bcl11a^ep−/−^* mice (Figure 7 and Figure 8b). Parallel confirmation came from the Day 10 immunoblot analysis, which showed similar pattern of reduced K6, K14, and K10 expression upon epidermal *Bcl11a* deletion (Figure 8a,c). Above observation further indicated rapid re-epithelialization and subsequent re-establishment of homeostasis in the *Bcl11a^ep−/−^* mice post wounding. 

## 4. Discussion

Cutaneous wound healing is a highly dynamic process involving complex crosstalk at both cellular and genetic level to facilitate timely commencement, transition, and smooth propagation of the injury response pathway, which can lead to efficient repair and ultimate restoration of skin homeostasis [10,11,13,14,15,16]. *Bcl11a* is a crucial developmental gene with mice harboring germline deletion (*Bcl11a^−/−^* mice) exhibiting perinatal lethality. A study by Li et al. (2017) showed EPB defects in murine embryonic skin null for BCL11A owing to disrupted epidermal differentiation and significantly altered lipid composition [29]. Results established its crucial role as a key skin morphogenesis regulator [29]. The present study establishes for the first time an important role of epidermal BCL11A in skin homeostasis and as a negative regulator of cutaneous wound healing. The epidermis specific *Bcl11a* knockout mice (*Bcl11a^ep−/−^* mice) serves as a prototype to gain mechanistic insights of various downstream pathways converging towards the manifestation of an accelerated healing phenotype in absence of BCL11A.

We initially determined the effects of epidermis specific *Bcl11a* excision on adult skin homeostasis and an in-depth analysis of epidermal markers showed significant changes between wild type and mutant skin at specific timepoints. A modest increase in epidermal differentiation (K10) was evident in *Bcl11a^ep−/−^* skin as early as P34 without any discernable difference in expression of markers associated with epidermal proliferation (K14, PCNA) between *Bcl11a^L2/L2^* and *Bcl11a^ep−/−^* mice (data not shown). These changes in epidermal proliferation and differentiation became more pronounced with advancing age as confirmed by increased expression of epidermal basal marker K14, proliferation marker PCNA and early differentiation marker K10 in *Bcl11a^ep−/−^* mice on P39. Additionally, CLAUDIN-1 and OCCLUDIN are important tight junction proteins associated with proliferation and differentiation and in maintaining EPB homeostasis [50]. A previous study by Li et al. (2017) reported altered expression of genes encoding for junctional proteins (JP) in *Bcl11a^−/−^* mice, bringing into light CLAUDIN-1 as the most significantly downregulated JP in the mutant and a potential target of BCL11A [29]. Even though we did not observe a significant change in CLAUDIN-1 expression in *Bcl11a^ep−/−^* mice compared to the *Bcl11a^L2/L2^* on P34, the appreciable downregulation in CLAUDIN-1 expression observed at later timepoint in P39 *Bcl11a^ep−/−^* mice was coherent with our earlier observations made in *Bcl11a-null* mice (data not shown). No significant difference in the baseline expression of another JP OCCLUDIN was noted between *Bcl11a^L2/L2^* and *Bcl11a^ep−/−^* mice on either day (data not shown), further hinting towards a more specialized role of epidermal *Bcl11a* in selectively modulating expression of specific JP (CLAUDIN-1 in this case) and their relevance in maintaining normal skin morphology. Overall, the above results clearly indicate regulatory control of skin epidermal homeostasis by BCL11A at multiple levels.

*Bcl11a* is a well-known proto-oncogene with BCL11A overexpression found to be associated with enhanced invasion and metastasis across multiple cancer types such as breast cancers, hematological malignancies, glioblastoma, or laryngeal squamous cell carcinoma [26,51,52,53]. Interestingly, no discernible skin phenotypic changes were detected in *Bcl11a^ep−/−^* mice other than modest elevated differentiation accompanying elevated level of proliferation to maintain homeostasis. Additionally, the contrasting phenotype of barrier defect observed in embryonic *Bcl11a* null mice skin owing to reduced epidermal differentiation and altered lipid composition [29] and that of enhanced proliferation and differentiation in adult *Bcl11a^ep−/−^* mice skin further highlights the development stage specific functional divergence of epidermal BCL11A. Future analysis of certain key parameters such as a comprehensive view of the skin lipid composition of adult *Bcl11a^ep−/−^* mice, will enable us better to understand the mechanisms contributing towards increased epidermal proliferation and differentiation in absence of epidermal BCL11A.

Next, we analyzed the effects of epidermal *Bcl11a* deletion on cutaneous wound healing. The absence of BCL11A in the epidermis exhibited accelerated healing kinetics manifested in form of significantly enhanced wound closure rates starting Day 5 till the point of near complete closure on Day 10. Accelerated re-epithelialization was observed in the Day 8 wound bed of *Bcl11a^ep−/−^* mice potentially due to rapid proliferation and concomitant migration of keratinocytes in absence of epidermal BCL11A, coherent with its effects seen on unwounded skin [17,21,37,43,54]. The absence of hyperproliferative epithelium at Day 10 in *Bcl11a^ep−/−^* mice lacking BCL11A clearly showed its early commitment to differentiation and subsequent sloughing of the outer terminally differentiated cornified layers to restore homeostasis, which was yet to be achieved in the wild type *Bcl11a^L2/L2^* mice that exhibited persistent hyperproliferative epithelium even on Day 10 post wounding [47,55].

We further evaluated the influence of epidermal *Bcl11a* ablation on the activation, re-epithelialization and contraction phase of healing at Days 8 and 10 post wounding.

We first determined the cell autonomous effect of epidermal *Bcl11a* excision by studying their impact on stages of wound healing typically involving epidermal keratinocytes. One of the earliest events triggered upon injury is keratinocyte activation, wherein the keratinocytes in the supra-basal compartment adjacent to the wound edges become activated and start undergoing morphological changes and expressing K6 [21,56]. Re-epithelialization occurs during the later phase of healing post granulation tissue formation and involves regeneration of denuded epidermis [15]. The re-epithelialization phase of a healing cascade typically involves multiple proliferative epidermal cell types such as mitotically active/inactive K14 positive basal epidermal cells or dedifferentiated GATA6 and K14 positive epidermal cells being recruited from the junctional zone (JN) to regenerate the denuded epidermis [57], in turn providing a much more global view of the In Vivo re-epithelization process. PCNA, on the other hand, is only expressed by actively proliferative cells in the early G1 and S phase of the cell cycle [35,43,44]. The IHC and immunoblot analysis of the Day 8 wound biopsies harvested from *Bcl11a^L2/L^*^2^ v/s *Bcl11a^ep−/−^* mice showed significant downregulation of keratinocyte activation marker K6 and concomitant upregulation of K14 and K10 in *Bcl11a^ep−/−^* mice. By contrast, the Day 10 wound biopsies exhibited significant downregulation of all markers including K6, K14, PCNA and K10 in *Bcl11a^ep−/−^* mice. All the above results suggest early resolution of keratinocyte activation, rapid re-epithelialization and accelerated restoration of homeostasis in absence of epidermal BCL11A [14,33]. 

We also analyzed the non-cell autonomous effects of epidermal *Bcl11a* excision, if any, by analyzing cellular processes during wound healing not involving epidermal cells. Angiogenesis and myofibroblast mediated wound contraction are two key processes central to efficient wound healing, neither of which involves epidermal cells. The key players in angiogenesis are endothelial cells responsible for forming the blood vessels with the structural integrity being provided by pericytes residing in basal lamina to restore oxygen and nutrient supply to the wound bed [15]. The increased number and size of CD31 positive endothelial cells lining blood vessels in the Day 8 wound bed samples of *Bcl11a^ep−/−^* mice strongly justified advanced angiogenesis potentially through early commitment of the endothelial cells to growth and differentiation program [58]. The control *Bcl11a^L2/L2^* mice on the other hand, however showed smaller and scattered CD31 positive individual endothelial cells likely in the initial stages of vessel formation as would be expected in wild type mice with functional BCL11A. The process of fibroplasia occurs in two stages with the initial phase involving the formation of the granulation tissue and the later phase observing the differentiation of fibroblasts to myofibroblasts. The myofibroblast mediated wound contraction is primarily engaged in drawing wound margins together to enable prompt closure [48,59]. Chronic persistence of activated myofibroblasts leads to fibrosis, a form of pathological healing [60]. Even though overall estimation of the relative abundance of myofibroblasts in the Day 8 wound bed (by analyzing area fraction positive for α-SMA) exhibited a trend of decline in the *Bcl11a^ep−/−^* mice, the activated individual myofibroblasts in the mutant group showed a more elongated and stellate morphology compared to the circular shape predominantly observed in the control group. This relatively sparse yet elongated morphology of the myofibroblasts observed in the *Bcl11a^ep−/−^* Day 8 wound bed potentially suggests advanced maturation accompanying the concluding stages of the contractual phase of healing, additionally marked by the apoptosis of the mature myofibroblasts that have already finished their work as mentioned in literature [60]. This is yet to be achieved in the control group undergoing active contraction and hence showing higher abundance.

However, the impact of epidermal *Bcl11a* deletion on immune cell behavior during the inflammatory phase of healing has not been addressed in this study. Future studies analyzing the relative abundance of neutrophils, monocytes/tissue activated macrophages or adaptive immune system components such as Langerhans, dermal dendritic cells, and T-cells at various timepoints post wounding in *Bcl11a^L2/L^*^2^ v/s *Bcl11a^ep−/−^* mice would help us comment on its non-cell autonomous effect on the host response program elicited immediately following an injury [15]. Additionally, BCL11A regulated paracrine factors that could possibly be mediating its non-cell autonomous effects, such as on the angiogenesis program during wound healing, are yet to be identified. 

A variety of TFs serve as integration hubs, relaying ordered changes in the physiological level of proteins in response to external stimuli such as mechanical injury, radiation, or temperature induced damage to restore homeostasis [20]. Over the years, multiple TFs have been found relevant in the context of EPB homeostasis and wound healing such as JUN, JUNB, E2F1, E2F4, ETS2, MYC, BCL11B, FOS, FOXN1, KLF4, GATA3 and TP63 [21,24,29,61,62,63,64,65,66,67,68,69]. Studies have established the pleotropic nature of TFs in regulating multiple stages of healing through their ability to alter chromatin structure around target genes by associating with chromatin modifying complexes [70]. Identifying the expression of histone modifying enzymes and other regulatory proteins in skin of *Bcl11a^ep−/−^* mice and determining their possible interactions with epidermal BCL11A can help us to better determine the BCL11A governed transcriptional network regulating healing. 

A notable observation that came into light during our study is a modest upregulation in the expression of transcription factor BCL11B, a homolog of BCL11A and a related C2H2 zinc finger protein, in *Bcl11a^ep−/−^* mice at P34 (data not shown). Earlier work has strongly substantiated the role of BCL11B in EPB homeostasis, hair morphogenesis and cutaneous wound healing [21,32,71,72,73,74]. More specifically, a 2012 study by Bhattacharya et al. showed how mice selectively lacking BCL11B in the epidermis exhibits delayed wound healing subject to its impact on cell migration, proliferation, differentiation and HFSC pool in a cell autonomous manner and on granulation tissue formation/tissue contraction in a non-cell autonomous manner [21]. Additional studies are necessary to determine whether a compensatory upregulation observed in BCL11B expression in absence of BCL11A is a contributor towards the accelerated healing phenotype. Furthermore, noncoding RNAs such as microRNAs (miRNA) and long noncoding RNAs (lncRNA) have received significant attention recently, owing to their rapidly emerging role in modulating nearly every stage of a healing cascade [75,76,77,78,79]. The characterization of expression of noncoding RNAs in the *Bcl11a^ep−/−^* mutant skin might provide additional cues about its mechanistic role in facilitating healing. 

Li et al. (2017) previously identified AP-1 TF, Fos-related antigen 2 (FOSL2) and a lipid metabolism related protein for elongation of very long chain fatty acids protein 4 (ELOVL4) as transcriptional targets of BCL11A in regulating embryonic skin development [29,80,81]. Our immunoblot analysis on P34 samples showed similar trend in downregulation of FOSL2 and ELOVL4 expression in *Bcl11a^ep−/−^* versus *Bcl11a^L2/L2^* mice as reported in the *Bcl11a^−/−^* null mice (Supplementary Figure S2a,b). We also observed a consistent pattern of FOSL2 downregulation on the Day 10 skin wounds in *Bcl11a^ep−/−^* mice (data not shown). Earlier studies have documented a crucial role of FOSL2 overexpression in metastatic cancer progression across multiple cancer subtypes owing to its influence on cell migration and proliferation [82,83,84]. FOSL2 deficiency has been mostly correlated with tumor suppression, validating its potential as a possible therapeutic target [85,86]. Also, mice overexpressing FOSL2 have been reported to exhibit autoimmune T-cell mediated systemic inflammation which can further delay the healing process, further validating the relevance of FOSL2 downregulation in the accelerated *Bcl11a^ep−/−^* healing model [87]. Future studies targeted towards understanding the mechanistic regulation of these and other targets by BCL11A (either direct or indirect) and their impact on specific stages of healing will help us determine whether such factors e.g., FOSL2 and ELOVL4 contributes to the accelerated healing in *Bcl11a^ep−/−^* mice.

In conclusion, the current study reports a striking and a novel role of epidermal BCL11A as a negative regulator of cutaneous wound healing. No gender based significant differences were observed between the mutant and the control groups. It also elaborates on the cell or non-cell autonomous role of epidermis specific *Bcl11a* deletion in modulating distinct phases of healing such as keratinocyte activation, angiogenesis, and re-epithelialization with its final manifestation in the form of an accelerated healing phenotype. The persistent results obtained at two different timepoints of study (Day 8 and Day 10) portray the effects of epidermal *Bcl11a* deletion during the entire process and further highlights the multifaceted regulation by epidermal BCL11A during physiological cutaneous wound healing. However, the detailed underlying molecular mechanisms of the physiological function of an evolutionary selected protein such as BCL11A in mediating skin tissue regeneration, healing and restoration remains to be determined. 

Additionally, while the presence of the thin muscular layer (the *panniculus carnosus*) in mice facilitates healing via contraction, human skin being devoid of this layer primarily heals through granulation tissue formation and re-epithelialization [88]. This present study addresses the role of epidermal *Bcl11a* deletion in the context of healing both via epithelialization (~50%) and contraction (~50%) in a murine excisional wound healing model [89]. Future studies involving splint-wound models might provide additional insights on the BCL11A function in relation to cutaneous healing in humans that is primarily based on epithelialization [88,90]. Moreover, future multi-omics (transcriptomic, proteomic and metabolomic) will allow us to identify the BCL11A target genes, the corresponding proteins that are key in these cellular processes, and to pinpoint the mechanistic basis underlying this accelerated healing process triggered in absence of epidermal BCL11A. The unique potential of epidermal BCL11A brought forth in this study in accelerating healing kinetics without hampering normal skin homeostasis can serve as a great future tool to treat chronic and lingering wounds by selective manipulation of its expression either at the gene level utilizing gene-silencing strategies or at the protein level via topical application of a neutralizing antibody [91,92].

## Data Availability

The data that support the findings of this study can be made available upon request by the corresponding author as per the institutional guidelines.

## Supplementary Materials

The following supporting information can be downloaded at: https://www.mdpi.com/article/10.3390/cells11132106/s1, Figure S1: Immunoblot analyses of K14, PCNA, and K10 expression in P39 tail skin epidermal protein extracts; Figure S2: Immunoblot analyses of BCL11A targets (FOSL2 and ELOVL4) in P34 tail skin epidermal protein extracts.; Table S1: List of primers used for genotyping; Table S2: List of antibodies used for immunoblotting (IB); Table S3: List of antibodies used for immunohistochemistry (IHC).
