# Supplementary material for: Selective Ablation of BCL11A in Epidermal Keratinocytes Alters Skin Homeostasis and Accelerates Excisional Wound Healing In Vivo
Source: Cells. 2022 Jul 3;11(13):2106. doi: 10.3390/cells11132106 (PMC9265695; doi:10.3390/cells11132106)
Supplement: Supplementary file 1 [file cells-11-02106-s001.zip › cells-1769720-supplementary.pdf]

Supplementary Figure1

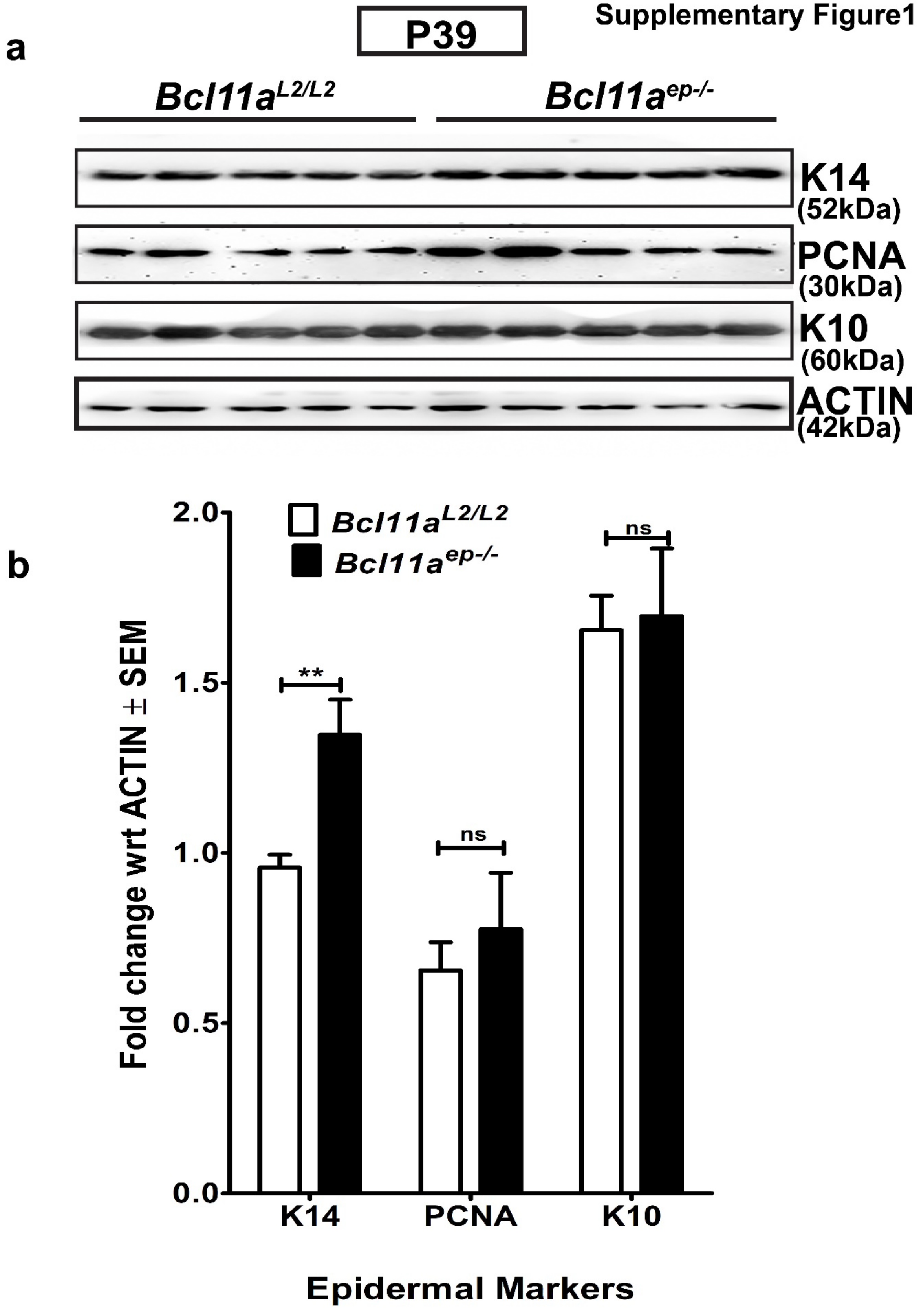

Supplementary Figure S1: **(a)** Immunoblot analyses of basal keratinocyte marker (K14), active proliferation marker (PCNA), and early differentiation marker (K10) expression in epidermal protein extracts isolated from P39 tail skin samples. β-actin was used as the internal control. **(b)** Bar graph quantification displaying the relative fold change in the expression status of epidermal markers (K14, PCNA and K10) in P39 *Bcl11a*$^{L2/L2}$ versus *Bcl11a*$^{ep-/-}$ tail epidermal extracts. N=5. N represents the number of animals of each genotype (*Bcl11a*$^{L2/L2}$ or *Bcl11a*$^{ep-/-}$) being involved in the study. **P<0.01, ^{ns}<non-significant.

Supplementary Figure2

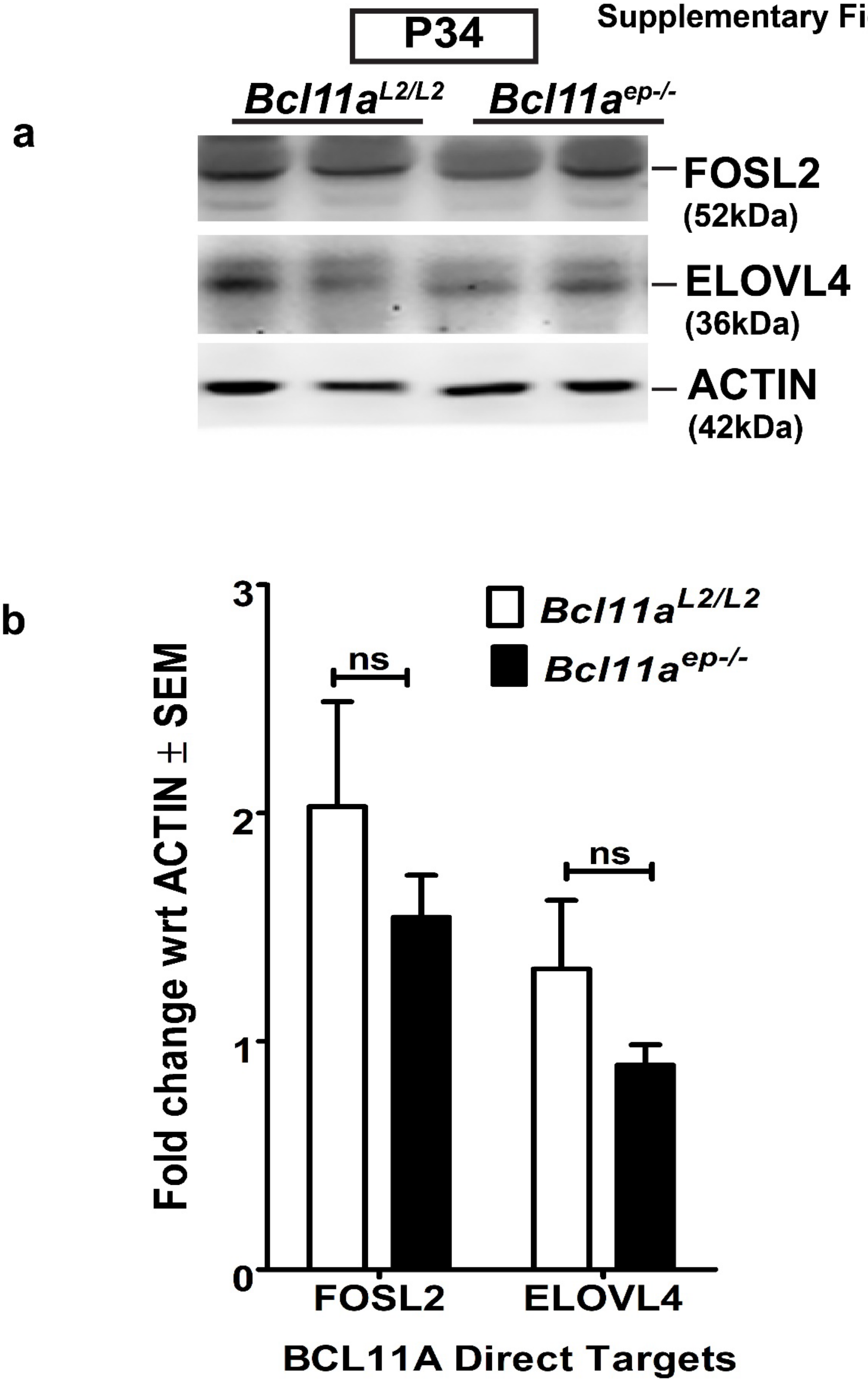

Supplementary Figure S2: **(a)** Immunoblot analyses of previously identified BCL11A targets (FOSL2 and ELOVL4) in epidermal protein extracts isolated from P34 tail skin samples. β-actin was used as the internal control. **(b)** Bar graph quantification displaying the relative fold change in the expression status in P34 *Bcl11a*$^{L2/L2}$ versus *Bcl11a*$^{ep-/-}$ tail epidermal extracts. N=4. N represents the number of animals of each genotype (*Bcl11a*$^{L2/L2}$ or *Bcl11a*$^{ep-/-}$) being involved in the study. ^{ns}<non-significant.

SUPPLEMENTAL TABLES S1-S3

Table S1: List of primers used for genotyping

Gene	Strand	Sequence
Bcl11a$^{L2/L2}$ (*L2*)	Forward	5’-CCCAGAGTAGCAAGCTCACC-3’
	Reverse	5’-CACTCGATCACTGTGCCATT-3’
Bcl11a$^{ep-/-}$ (*L*-)	Forward	5’-CCCTTGACTCTTTGGCTTGA-3’
	Reverse	5’-CACTCGATCACTGTGCCATT-3’
Cre	Forward	5’-ATTTGCCTGCATTACCGGTC-3’
	Reverse	5’-ATCAACGTTTTCTTTTCGG-3’

Table S2: List of antibodies used for Immunoblotting (IB)

Antibody	Type	Working Dilution	Company	Catalog Number	Host
K10	Primary	1:3333	Biolegend	905401	Rabbit
PCNA	Primary	1:2000	Abcam	Ab29	Mouse
K6	Primary	1:1000	Santa Cruz Biotechnology	Sc-56373	Mouse
ACTIN	Primary	1:2000	Bethyl Laboratories	A300-491A	Rabbit
K14	Primary	1:15000	Biolegend	905301	Rabbit
FOSL2	Primary	1:83	DSHB	PCRP-FOSL2-1B1	Mouse
ELOVL4	Primary	1:500	Novus Biologicals	NBP1-68493	Rabbit
Goat Anti Rabbit H&L Chain	Secondary	1:10000	Calbiochem	401315	Goat
Goat Anti Mouse H&L Chain	Secondary	1:10000	Calbiochem	401753	Goat

Table S3: List of antibodies used for Immunohistochemistry (IHC)

Antibody	Type	Working Dilution	Company	Catalog Number	Host
K10	Primary	1:500	Biolegend	905401	Rabbit
PCNA	Primary	1:6000	Abcam	Ab29	Mouse

K14	Primary	1:500	Biolegend	905301	Rabbit
K6	Primary	1:200	Santa Cruz Biotechnology	Sc-56373	Mouse
CD31	Primary	1:50	Abcam	Ab28364	Rabbit
α-SMA	Primary	1:100	Biolegend	904601	Mouse
CY^{TM} 3-Conjugated AffiniPure Goat Anti Mouse IgG (H+L)	Secondary	1:400	Jackson Immunoresearch	111-225-144	Goat
CY^{TM} 2-Conjugated AffiniPure Goat Anti Rabbit IgG (H+L)	Secondary	1:600	Jackson Immunoresearch	115-165-003	Goat
